# Promotion of Self-Management of Chronic Disease in Children and Teenagers: Scoping Review

**DOI:** 10.3390/healthcare9121642

**Published:** 2021-11-27

**Authors:** Marta Catarino, Zaida Charepe, Constança Festas

**Affiliations:** 1 Health Department, Polytechnic Institute of Beja, 7800-111 Beja, Portugal; 2 Institute of Health Sciences (ICS), Universidade Católica Portuguesa, 1649-023 Lisboa, Portugal; zaidacharepe@ucp.pt; 3 Center for Interdisciplinary Research in Health (CIIS), Institute of Health Sciences (ICS), Universidade Católica Portuguesa, 1649-023 Lisboa, Portugal; cfestas@ucp.pt; 4 Institute of Health Sciences (ICS), Universidade Católica Portuguesa, 4169-005 Porto, Portugal

**Keywords:** self-management, chronic disease, children, teenager, health education, pediatrics

## Abstract

Background: The scientific literature describes that self-management of chronic illness leads to improved health outcomes. Knowledge about interventions that promote self-management behaviors in children and teenagers has been poorly clarified. This study aims to map, in the scientific literature, the nature and extent of interventions that promote self-management of chronic disease, implemented and evaluated in contexts of health care provided to children and teenagers. Methods: The guidelines proposed by the Joanna Briggs Institute were followed. The survey was conducted in June 2021, with access to international databases and gray literature, in Portuguese, English, French, and Spanish. Results: Interventions that promote self-management of children and teenagers can be developed through a local contact or through technological means of support for health care. The use of online supports, such as applications or communication platforms, should be parameterized with health professionals, according to the needs of users. Conclusions: The acquisition of self-management skills in pediatrics is a process supported by the family, health professionals and the community, in which the nurse, in partnership, can promote communication and health education through cognitive strategies, behavioral programs included in physical or online programs, adjusted to the patients’ needs.

## 1. Introduction

Since the beginning of the 20th century, scientific and technological innovations in neonatal and pediatric health care have led to evident changes in the epidemiology of health conditions at these stages of the life cycle. Infectious diseases have decreased substantially and life expectancy has increased, reflected in an increased prevalence of chronic disease [1], with associated physical and psychological comorbidities [2,3]. Up to 60% of these children have at least one concomitant psychological disorder [3].

Despite the changing pattern of illness, healthcare continues to be directed towards the treatment of acute illness and healthcare expenditure associated with chronic illness is increasing [4,5]. The accountability of people in the control of their own health [6] and a transfer of health care to non-hospital domains could be important strategies in the face of this reality [7].

Self-management is recognized as a public health intervention and an important component of health care, in all age groups [8,9,10]. It is considered a multidimensional and complex phenomenon, as it requires a variety of actions, in a wide variety of patients and conditions [1,7]. It has been applied to health promotion activities, as well as activities related to acute or chronic illness; however there is more evidence of its relationship with chronic illness [11,12].

According to the theory of individual and family self-management, which explores the self-management process components, these actions should involve patients as dyads, within the family or in the family unit as a whole and at all stages of development [7]. In turn, the pediatric self-management model adds that self-management behaviors require the involvement of the patient, family, community and the healthcare system in a triadic perspective [11,13,14].

The aiming of acquiring the capacity for self-management of the disease is to improve health behavior, reduce admission to health services and, consequently, improve the patient’s quality of life. It can be a challenge for them to acquire the ability to self-manage their disease, with little or no additional ongoing support, so it is essential to find support strategies to provide and promote this competence [15].

Although the world scientific literature identifies promising results, regarding the self-management of chronic illness in the pediatric population, there is little consensus on the procedural components [16]. In this population, this phenomenon is more complex, as over the life cycle roles change, family balance changes, information and support needs vary according to age and stage of development [7,17,18].

Promoting self-management in children/adolescents constitutes a real challenge to nursing practice. Nurses manifest the need to identify interventions that enable the acquisition of knowledge, skills and social facilitation for self-management of health in pediatrics [7,16], in order to achieve a positive impact on the health of this population [19].

In this domain, there is a need for a scope review to map the studies carried out on the self-management of chronic illness in children and adolescents in the context of health care in the scientific literature.

A preliminary search was carried out in the MEDLINE Complete, The Cochrane Database of Systematic Reviews and JBI Evidence Synthesis databases. No systematic literature reviews or scope reviews published on this topic were identified. This study will synthesize the identified interventions and provide a conceptual clarification that will allow nurses to base the operationality of interventions that promote self-management of chronic illness in children and adolescents.

## 2. Materials and Methods

The review was conducted according to the scoping review methodology and it was based on the methodological procedures defined by Joanna Briggs Institute (JBI), as it was described in JBI Reviewer’s Manual [20]. Was registered on the Open Science Framework (OSF) platform.

### 2.1. Review Question

The review question emerged through the mnemonic P (population—child and teenager with chronic illness), C (concept—self-management) and C (context—health care): “What is the published scientific evidence about promoting interventions of self-management of chronic illness in children and adolescents in the context of health care?”.

In the construction of the study, the following sub-questions emerged:

(1) What are the uses of the concept of self-management of chronic illness in children and teenagers in the context of healthcare?
(2) What activities have been implemented and evaluated to promote self-management of children and teenagers in healthcare?
(3) What are the characteristics of this intervention (unique and/or related activities, name, resources, frequency, duration, and background)?
(4) Which health professionals promote self-management in children and teenagers?
(5) What outcomes were evaluated after the intervention?

### 2.2. Elegibility Criteria

#### 2.2.1. Participants

The review considered studies that included children and teenagers. This was followed by the definitions issued by the Convention on the Rights of the Child, which considers every individual under 18 years of age to be a child [21] and the World Health Organization, which defines a person aged between 10 and 19 as a child [22].

The research was limited to school age—first, second and third cycle, involving studies whose patients were between 6 and 18 years-old. No restrictions were applied for studies centered on specific demographic factors (e.g., gender, ethnicity).

#### 2.2.2. Concept

In this study, citations that addressed the impact of using interventions (programs) that promote self-management by children and adolescents were considered eligible. It was intended to know the applicability of the concept in the provision of health care to children and teenagers with chronic illness, considering the promotion of self-management as a health intervention, of a multidisciplinary nature.

#### 2.2.3. Context

Studies that addressed interventions focused on children and teenagers with chronic illnesses, involving their caregivers, inserted in the family environment and in the different contexts of health care (hospital and community care, mainly school health) were included.

#### 2.2.4. Type of Studies

Quantitative studies were incorporated into the research: descriptive, observational analytical, experimental and experimental analytical and qualitative studies, such as: documentary research, case study, ethnography, phenomenology; mixed studies, as well as gray literature.

Editorials, letters to the editor, and abstracts were excluded from the review.

### 2.3. Research Strategy

A complete search strategy was developed, using indexed terms, alternative terms, as well as truncations and the Boolean operators AND and OR. The research was carried out in the subject, title and abstract and keyword fields, on 4 June 2021. Thus, the following research equation was obtained: (“self-management*” OR “management self*” OR “self care*”) AND (child* OR adolescent* OR teen* OR youth* OR “young people” OR pediatric* OR pediatric* OR infant* OR scholar) AND (“chronic disease*” or OR “chronic illness*” OR “chronically ill*” OR “special health needs*” OR “special health care needs*” OR “special healthcare needs*” OR “complex chronic illness*” OR “complex chronic condition*” OR “complex chronic disorder*” OR “chronic condition*”) AND (“health” OR “delivery of health care” OR “healthcare delivery” OR “healthcare ”OR “health care”). Due to the language barrier, studies published in English, French, Spanish, and Portuguese were selected, without time limitations, in the following databases: Scopus by Elsevier, 1975–2021; Web Of Science by Clarivate, 1988–2021; Pubmed, 1991–2021; CINAHL Complete by EBSCO Host, 1992–2021.

Cochrane Central Register of Controlled Trials, by EBSCO 1996–2021, and Psychology & Behavioral Sciences Collection, via EBSCO Host (2002–2021).

The search of unpublished studies in the gray literature included the Scientific Open Access Scientific Repositories of Portugal (RCAAP) database.

The investigation strategy is described in detail in Appendix A (Table A1).

### 2.4. Selection of Studies

After the search, all identified citations were exported to the EndNote Web software (Clarivate Analytics, Philadelphia, PA, USA) [23]. Duplicates have been removed in this process.

The effective selection of studies was carried out in two phases, using the Rayyan QCR platform [24]. The first included reading their titles and abstracts and eliminating those that did not meet the eligibility criteria. This screening was performed by two investigators who independently assessed it against the inclusion criteria. In the second stage, the full text of the citations selected as potentially relevant was also evaluated in detail by the two independent investigators.

A total of 1893 studies were obtained in the search performed, in 4 June 2021: 364, from MEDLINE Complete, 215 from CINAHL, 741 from Scopus, 494 from Web of Science, 37 from Cochrane, 21 from Psychology & Behavioral Sciences Collection, and 16 from Scientific Open Access Scientific Repositories of Portugal (RCAAP). A total of 866 studies were excluded for identifying themselves in duplicate. Two independent researchers analyzed the remaining 977 articles. The title and abstract were reviewed and 706 studies were excluded, which allowed us to evaluate 271 articles for eligibility.

Studies for final review were selected by confirming the selection and exclusion criteria, after individual reviews and investigator meetings. 160 studies were excluded, whose content did not answer the initial question, 75 studies, whose age did not fall within the age range defined in the inclusion criteria; 8 studies were based on theoretical models, which contributed to the foundation of this construct, but not to data analysis; 7 articles, whose type of study also did not fit the eligibility criteria, especially review protocols.

**Figure 1:**
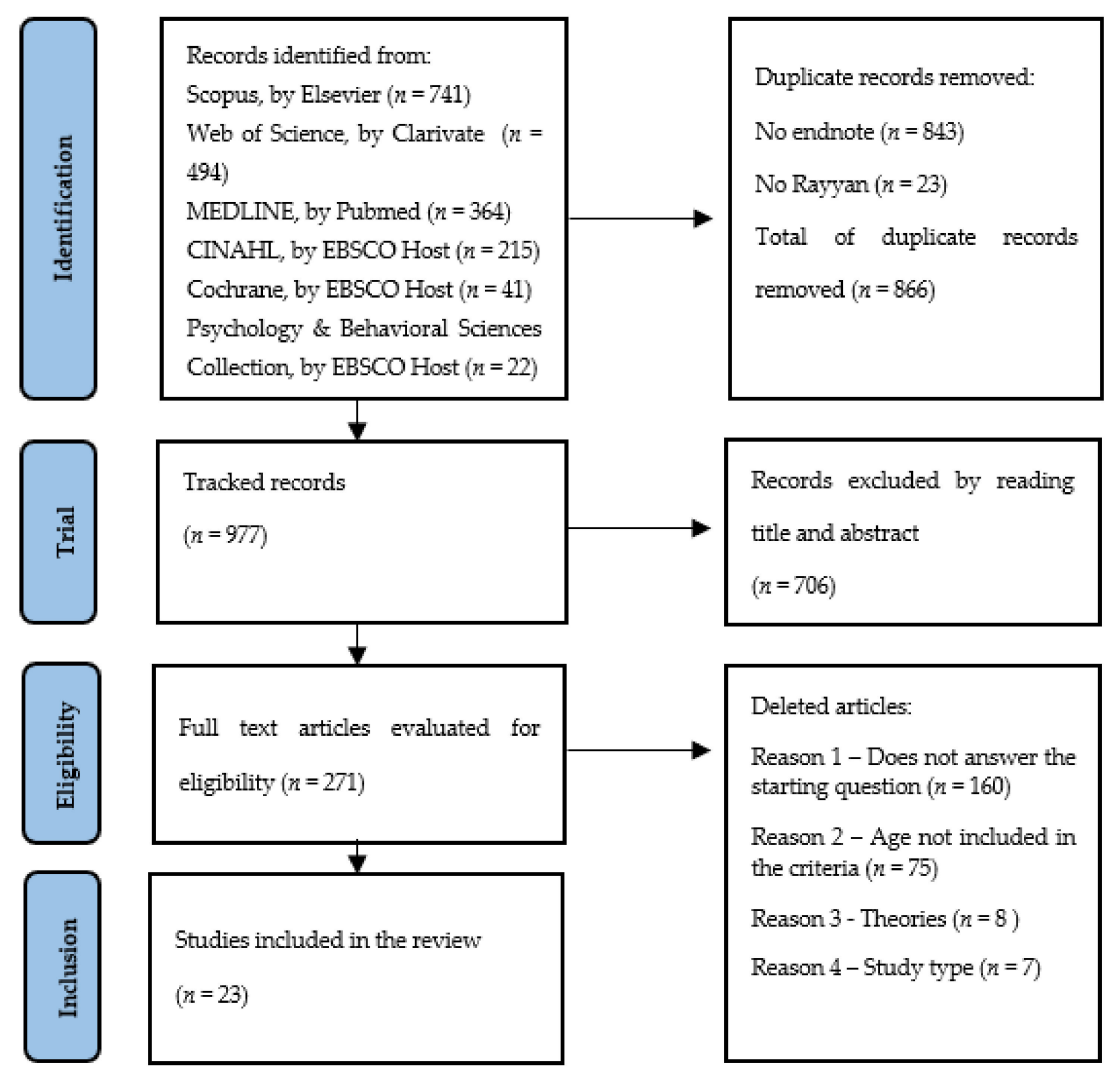
PRISMA of study selection and inclusion process [25].

A total of 23 studies were included in this analysis. The data collection and selection processes are shown in Figure 1.

### 2.5. Data Extraction

The data were extracted from the works included in the scoping review by two reviewers, independently. A structured instrument, defined by the JBI [26], (Table A2 in Appendix B) was used, where the following information was transferred: author/s, year, country, title, type of study, and population); the use of the concept in providing health care to children and adolescents; interventions implemented and evaluated to promote self-management of children and adolescents; its characteristics in this population; the health professionals who promote them and the results obtained after the interventions.

### 2.6. Data Analysis and Presentation

The collected data were synthesized in schematic and tabular form, facilitating mapping, as recommended by the JBI [25], by consensus of two investigators.

The information contained in the tabular presentation is complemented with a narrative summary, with a discussion of the results and a description of their relationship with the research objectives and questions.

## 3. Results

### 3.1. Characteristics of Included Studies

All articles selected in this scope review were published between 2000 and 2021, with 65.2% (*n* = 15) published in the last 10 years. All were published in the form of journal articles (100%). In 48% of the studies used a quantitative methodology (*n* = 11) and 39% used a qualitative methodology (*n* = 9). Studies that used mixed methods were also identified (*n* = 2.9%). Most of the population included in the studies were adolescents with chronic disease: asthma (*n* = 8), type 1 diabetes (*n* = 4), spina bifida (*n* = 3), cystic fibrosis (*n* = 2), kidney transplant recipients (*n* = 2), hematic cancer (*n* = 1), juvenile idiopathic arthritis (*n* = 1), migraine (*n* = 1), and an article whose adolescents had asthma and/or epilepsy (*n* = 1). Only 6 studies (26.1%) included participants under 10 years of age. All other studies were directed to populations from 11 and 12 years of age (*n* = 17, 73.9%). As for origin, the United States of America was the most represented country with 33.3% (*n* = 8), followed by Canada, with 4 studies, Netherlands 2, United Kingdom 2, Portugal, Italy, Israel, Germany, Argentina, India, South Korea, and Australia, with 1 study each. The main features of the articles included in this scoping review are additionally reported in Appendix C (Table A3).

### 3.2. Review Results

The interest in promoting self-management of chronic illness in children and adolescents in this context is represented in the literature, mainly in the nursing discipline.

**Scheme 1:**
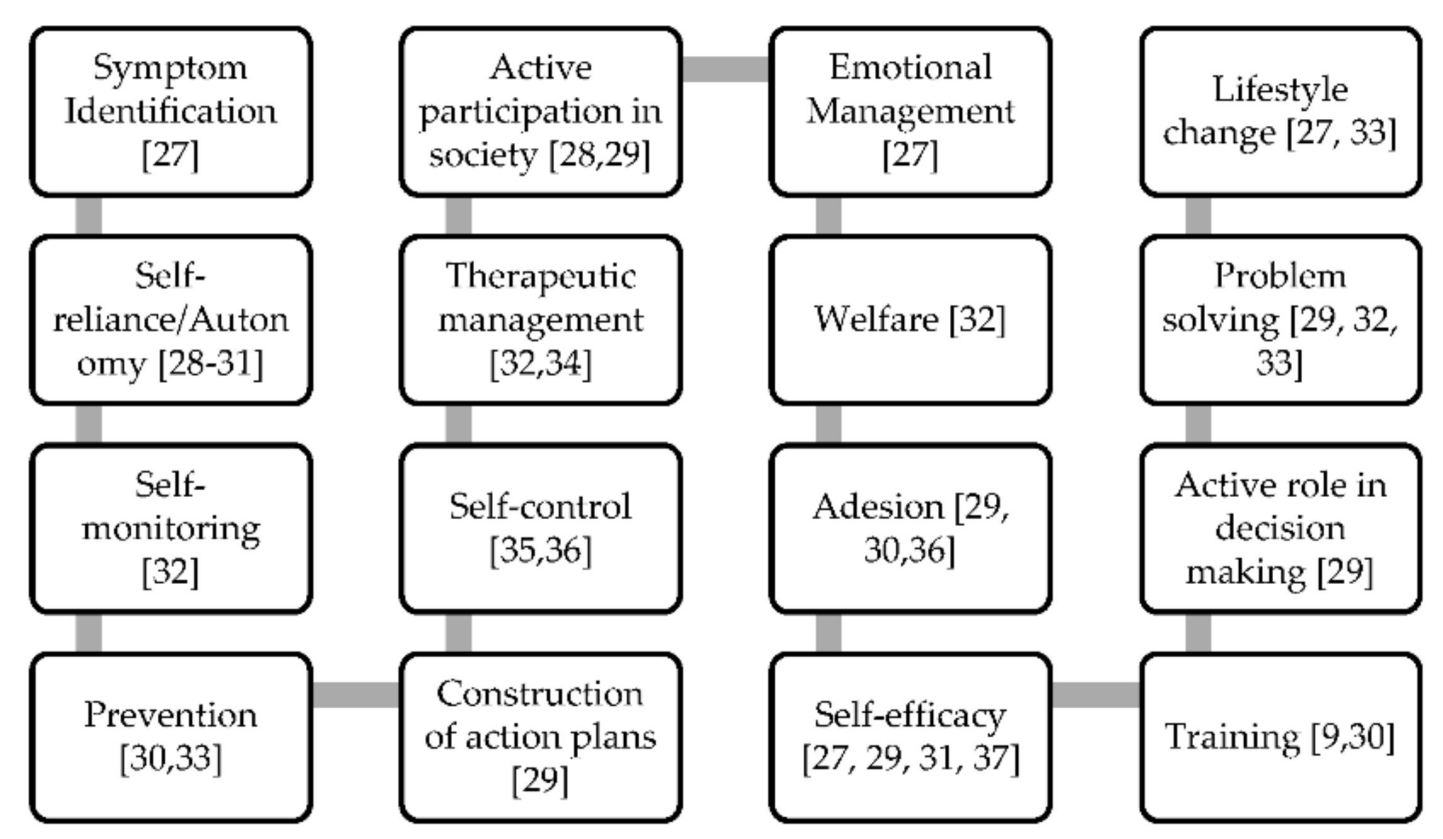
Uses of the concept of self-management of chronic illness in children and adolescents [9,27,28,29,30,31,32,33,34,35,36,37].

With regard to question 1 (What are the uses of the concept of self-management of chronic diseases in children and adolescents?), although all studies addressed the paediatric population, they mostly used a broad concept of self-management, not exclusively applied to this population. However, similarities are identified in the application of the concept with regard to self-management behaviours (Scheme 1).

One piece of data extracted from this study, which we consider relevant for the conceptualization of self-management of chronic illness in children and adolescents, was the fact that the studies generally specify the term support (*n* = 8, 35%) and support (*n* = 7, 30%) of self-management, referring to the fact that self-management in pediatrics is a supported process, using support strategies using parental or reference figures for children and adolescents.

Appendix C (Table A4) reported the data presentation template for Question 1.

**Scheme 2:**
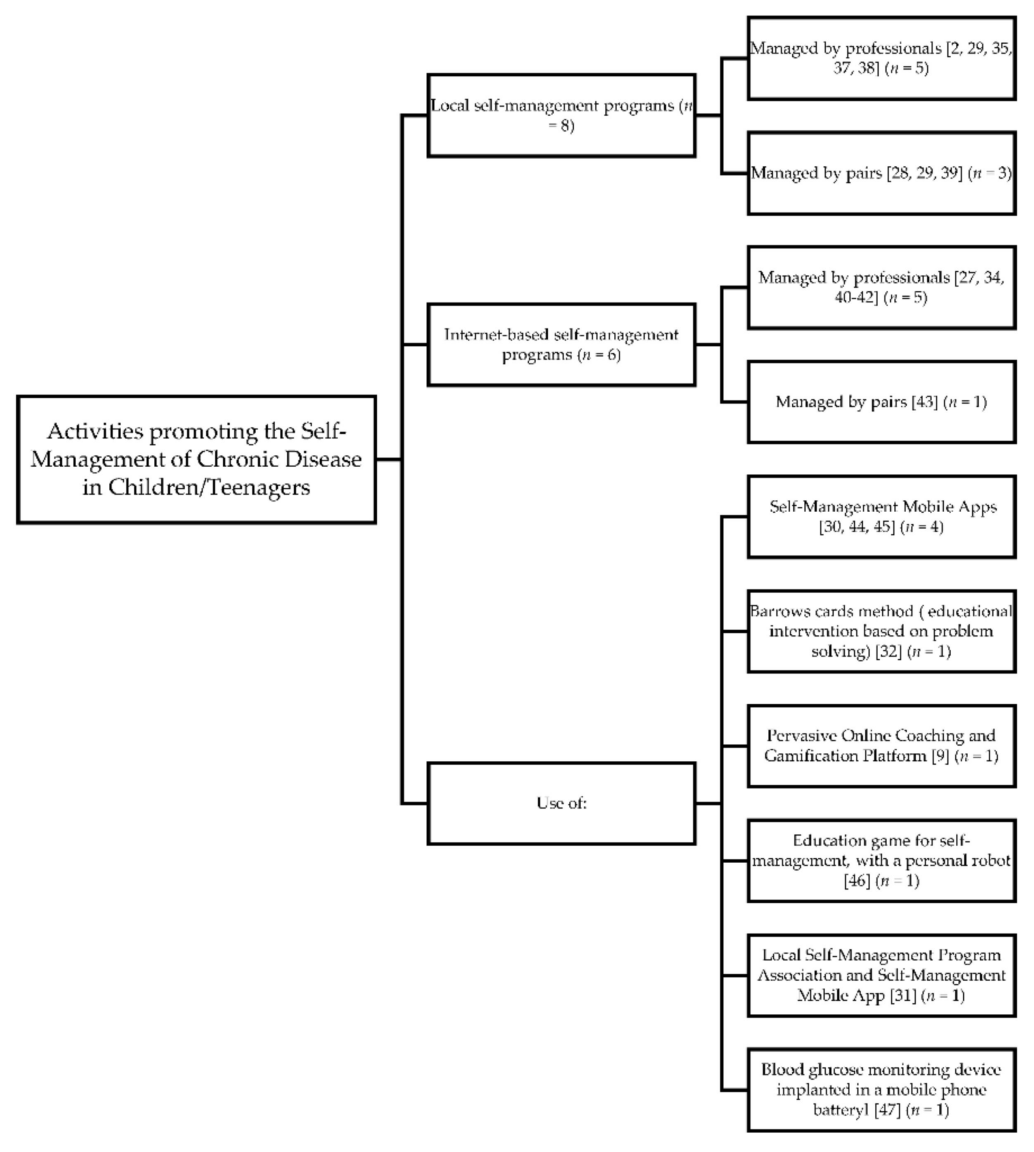
Activities related to the intervention to promote self-management of children and teenagers [2,9,27,28,29,30,31,32,33,34,35,37,38,39,40,41,42,43,44,45,46,47].

**Scheme 3:**
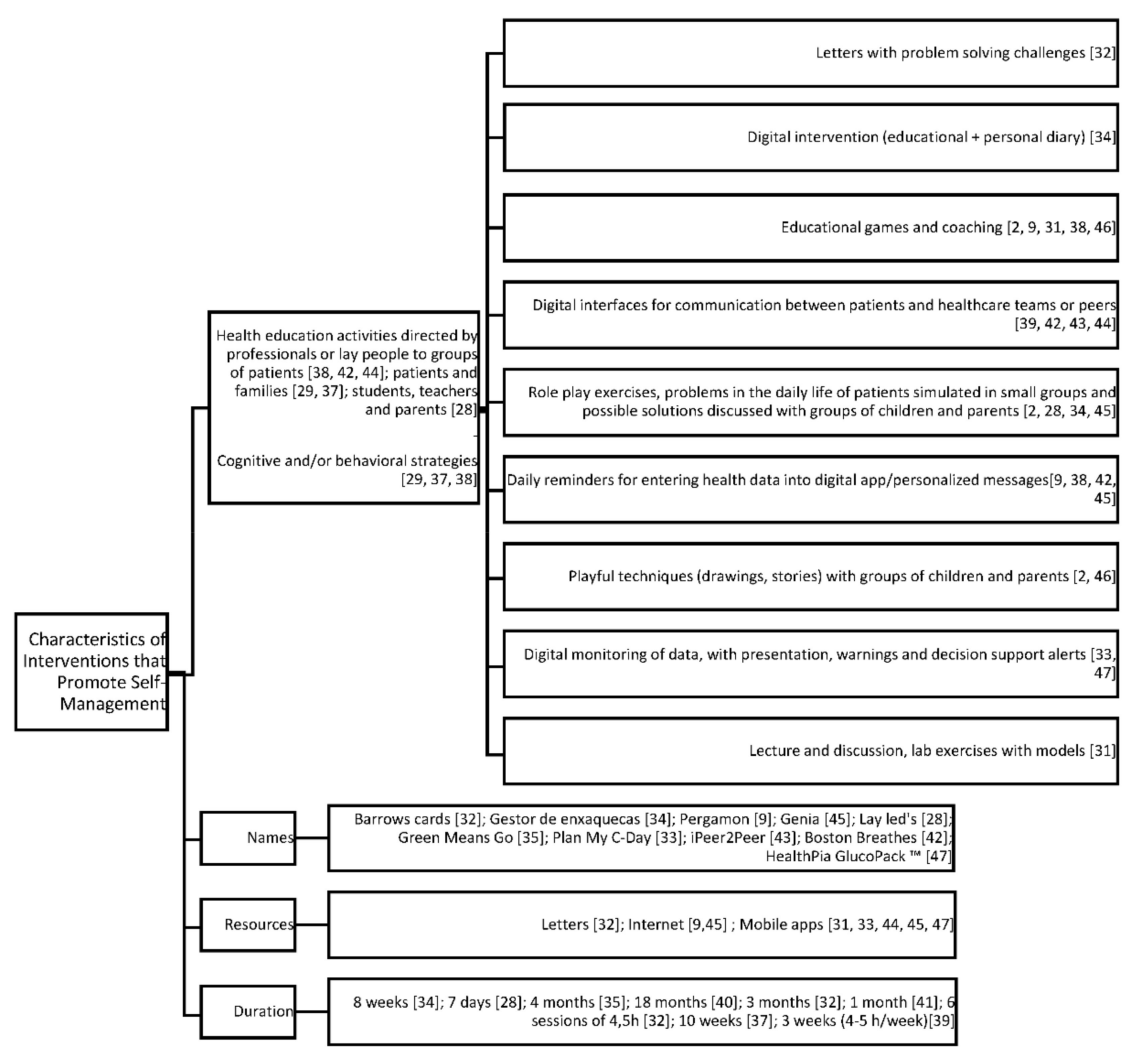
Characteristics of interventions that promote self-management of chronic illness in children and adolescents [2,9,28,29,31,32,33,34,35,37,38,39,40,41,42,43,44,45,46,47].

Regarding Question 2 (What activities were implemented and evaluated to promote self-management of children and adolescents in healthcare?), most studies analyzed activities developed through face-to-face self-management programs, which were taught in school context and summer camps (Scheme 2). These programs were run by health professionals (*n* = 7) or by laypersons (peers) (*n* = 3). This was followed by educational and interpersonal communication programs in an online format run by health professionals (*n* = 5) and by peers (*n* = 1). The use of mobile apps was also an activity typology reported in the studies (*n* = 4) (Scheme 3). Other activities were also identified, such as the use of the Barrow Cards method and digital strategies, such as the coaching platform and pervasive gamification–a game using a robot and the use of a digital monitoring device. Appendix C (Table A5) describes the data presentation model for Question 2.

Regarding Question 3 (What are the characteristics of these interventions (unique and/or related activities, name, resources, frequency, duration?), the identified interventions were covered by numerous types of face-to-face and virtual activities. When face-to-face, mostly in groups, from the use of cards with problem solving challenges; role play exercises; problems in the daily life of simulated patients (physical and virtual games, drawings, stories, videos, and dramatizations), with groups of children and parents; lectures and discussion; laboratory exercises with anatomical models; reminders for entering digital health data and personalized messages. Many of these activities use cognitive-behavioral strategies, based on establishing communication between patients and healthcare teams or patients and peers, respecting the goals of each patient The self-management education sessions were directed at groups of patients, families, students, teachers and parents. The duration of interventions ranged from 6 days to 18 months. Several names have been identified and are referenced in Scheme 3.

Appendix C (Table A6) reported the data presentation template for Question 3.

Regarding Question 4 (Which health professionals promote self-management in children and adolescents?), the results reinforce that many of the interventions are developed by multidisciplinary teams, however the studies identified the nursing intervention in 57% of studies (*n* = 13); medical intervention in 52% (*n* = 12); intervention by specialists in computer design, telemedicine, computer science, engineering in 17% (*n* = 4); physiotherapy or psychology 13% (*n* = 3); nutrition 19% (*n* = 2); and occupational therapy or social service intervention 4% (*n* = 1).

Appendix C (Table A7) describes a data presentation template for Question 4.

**Scheme 4:**
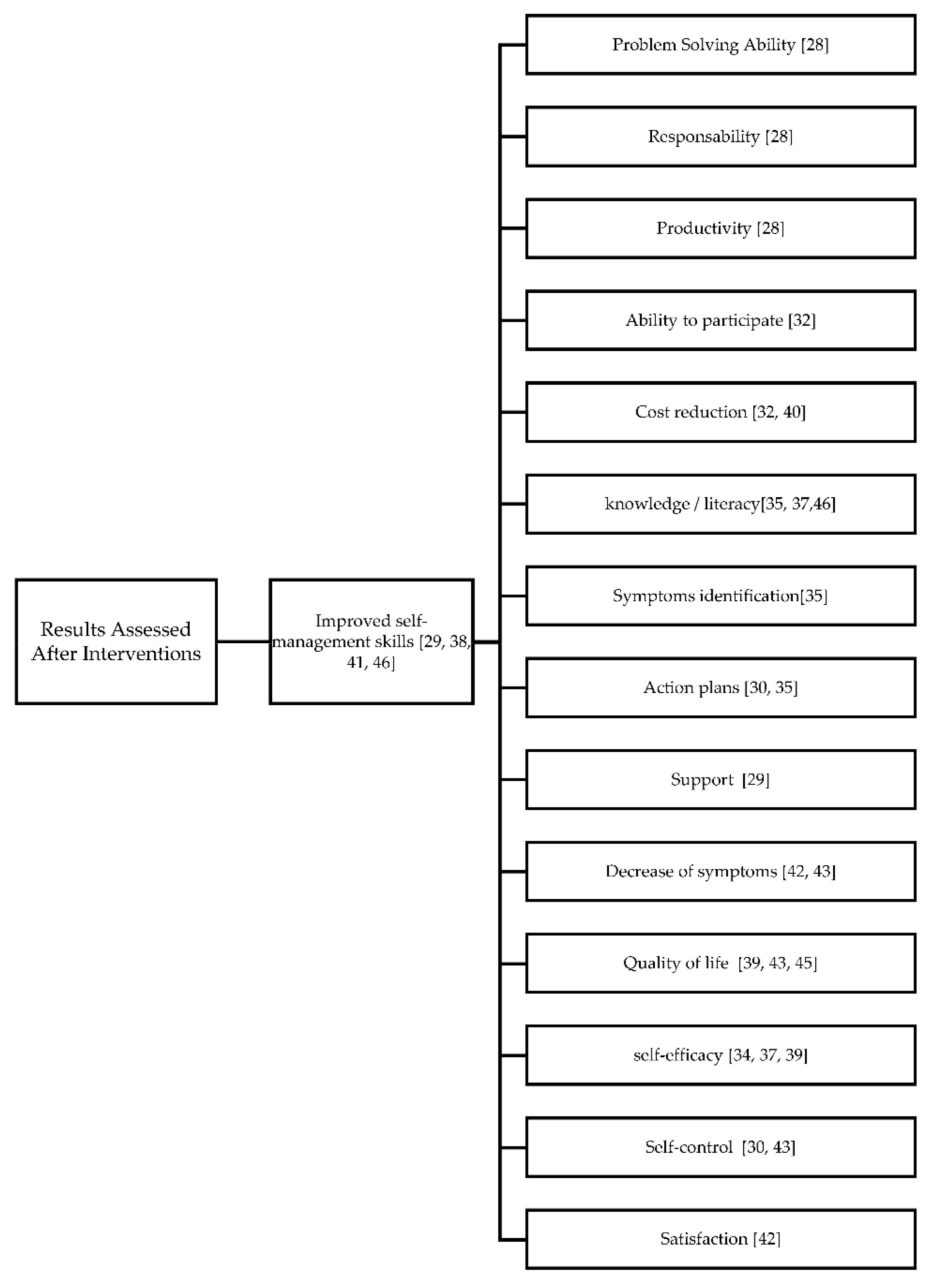
Results evaluated after interventions promoting self-management in children and adolescents with chronic illness [28,29,30,32,34,35,37,38,39,40,41,42,43,45,46].

Regarding Question 5 (What results were evaluated after the intervention?), several consequences or benefits were identified in the use of interventions that promote self-management, including problem-solving ability, responsibility, productivity, ability to adhere to treatment, reduction of health costs, knowledge/literacy, identification of symptoms, construction of action plans, support, reduction of symptoms, improvement in quality of life, self-efficacy, self-control, satisfaction (Scheme 4).

Appendix C (Table A8) describes the data presentation model for Question 5.

## 4. Discussion

There is a consensus that effective self-management can be achieved at an early age, enabling the prevention and management of diseases throughout life, with the development of the capacity to respond to daily challenges and solve problems [31,32]. Although the studies apply to populations of school age, from the age of 6 years, revealing that at this stage it is already possible for the child to develop a process of self-management skills, most studies are aimed at adolescents, at the stage of the life cycle in which there is a more evident progression towards the construction of their identity and autonomy. As this age group progresses in growth and development into adulthood, enhanced self-management skills and knowledge are essential to promote health and prevent problems [38].

Knowledge from the disciplines of nursing, medicine, psychology, physiotherapy, occupational therapy, nutrition, social work, and education can be integrated, using cognitive and/or behavioral strategies [27,33,37] as a basis for support to self-management of chronic illness in the child/teenager.

In the nursing discipline, these interventions represent, above all, health education, being the elementary school space in this approach. School-age children face many barriers to self-management in the school environment. Achieving self-management at this stage is important for maintaining health and quality of life into adulthood [31,47].

Self-management programs attempt to provide chronically ill patients with the knowledge, skills, and self-efficacy needed to take an active role in managing their health condition [29,33].

Nursing resources are not always sufficient, identifying in a study, the potential role of nurses in partnership models or support strategies, to provide schools with much-needed health resources [35]. In the analyzed studies, successful partnerships with nursing and medical students were identified [35]. Peer support, often in conjunction with family and teachers, was also an activity identified as positive [36,39,43]. 

In a study developed in the United States of America, in 19 classrooms where children with asthma were enrolled, 12 teachers (80%) participated in small group education sessions conducted by master’s degree students in nursing. In a second phase, sessions with the children and parents (48 students) and daily health records were developed, and 100% of the children were able to correctly verbalize the care to have with their specific condition, at the end of the study [35].

One study using Airways, a cystic fibrosis self-management program run by the child and caregiver using pen and paper records to aid decision-making, provided strategies to overcome barriers to the treatment of asthma. There was an increase in knowledge of asthma in all defined skill areas during the intervention period. Satisfaction was high, with 88% (14/16 participants) stating that they would continue to use the system [37].

Peer support interventions also include information-based programs, as well as cognitive and/or behavioral approaches aimed at increasing knowledge, self-confidence, or self-efficacy, as well as the use of self-care behaviors [27,28]. A study conducted on a holiday camp in Portugal on a program aimed at adolescents with spina bifida, by peers, revealed unanimous results and confirmed the importance of the program for the development of self-management activities. The psychoeducational intervention strategies used in the program (problem solving, role play, action plan, and modeling) were clearly demonstrated and may be associated with significant improvement in self-management behaviors [28].

In recent years, technology-based interventions to improve self-management have been shown to be useful in promoting self-management of chronic illness [9,29,30,33,44].

Patients with poor asthma control, in a study carried out in the Netherlands, were able to acquire the ability to self-manage their asthma through the internet (65%) [41]. High ratings were given in terms of the system’s ease of use and ease of learning through text messages. Compared with control patients, patients in the intervention group showed a significant improvement in adherence at 7 days, with a mean gain of 1 day of adherence and a median change of 4 to 6 days, compared to no median change in the control group [41].

One study using a Plan My C-Day mobile app specifically designed to promote cognitive self-management skills among adolescents with celiac disease had promising results. It demonstrated that content, resources and functions were operative in the process of self-management of the disease [33].

The app repositories include hundreds of apps claiming to improve self-management of illnesses, health outcomes, and health-related behaviors. However, there are few evidence-based solutions developed with the involvement of health professionals, patients and caregivers [44,45]. Most applications that collect patient-generated health data are not integrated into the care plan or clinical workflow. Without such integration, technologies can have only a minimal impact on the care and self-management of chronic diseases [44].

A Self-Management Platform for Children and Adolescents with Cystic Fibrosis (Genia) illustrated successful integration characteristics. The findings highlighted cultural characteristics of the clinical environment that are more likely to support the viable integration of new technologies as well as mHealth design components that contribute to successful self-management [44].

Although some support/support interventions promoting self-management show user acceptance and positive preliminary adoption of self-management practices, the field lacks detailed research that explores the perspective of users of these technologies [45].

## 5. Conclusions

Interventions that promote self-management of chronic illness in children and adolescents include activities, based on information, designed to achieve knowledge about the disease and the acquisition of skills. These presuppose a multidimensional and interprofessional support approach at a stage of the life cycle that requires a gradual adaptation, due to the development of cognitive and motor skills that takes place during this period.

Some of the activities mapped are aimed at children and adolescents themselves, but also at reference figures (family, teachers, health professionals), with the objective of conducting a process of supported self-management, according to personal needs (physical and emotional) of each patient. These activities are included in programs that use cognitive and/or behavioral strategies designed to increase self-confidence and self-efficacy.

Promoting chronic disease self-management emphasizes the role of patient education in preventive health care activities, disease self-monitoring, therapeutic management, action plan building, emotional management, treatment adherence, and patient control symptoms.

The nurse, in collaboration with other members of the multidisciplinary team, together with parents and teachers, may develop interventions conducted through direct contact, between members of this triad or through technological support means created for this purpose, provided that they are scientifically validated and constructed according to the needs of users.

## 6. Limitations/Future Prospects

Limitations were identified in some of the studies found. In some cases the sample of participants was small and the attrition rate relatively high. Studies were found in which the participants were not grouped according to the stage of development or age group, within the pediatric age, and there was a generalization of the findings.

Some studies did not use a pre-test and post-test design to measure the degree of change before and after completion of self-management programs.

Studies that evaluated only the acceptance of prototype versions, without the possibility of continuous usability of self-management support instruments, were identified.

We found cases where the users’ opinion about the interventions that promote self-management of chronic disease in children and adolescents was evaluated, but direct results, such as better self-management capacity, treatment adherence, were not evaluated. or quality of life.

In the future, research will be needed that takes into account all these factors.

## Appendix A

**Table A1:** Search strategy used in one of the databases.

Search	Query	Records Retrieved
#1	“Self-management*” [Mesh] OR “Self Management*” [Title/Abstract] OR “Management Self*” [Title/Abstract]	22,357
#2	Child* [Title/Abstract] OR Adolescen* [Title/Abstract] OR Teen* [Title/Abstract] OR Youth [Title/Abstract] OR “Young people” [Title/Abstract] OR Paediatric* [Title/Abstract] OR Pediatric* [Title/Abstract] OR Infant* [Title/Abstract] OR Infancy [Title/Abstract] OR Scholar [Title/Abstract] OR “Adolescent” [Mesh] OR “Child” [Mesh] OR “Infant” [Mesh]	4,271,039
#3	“Chronic Disease*” [Mesh] OR “Chronic Ilness” [Title/Abstract] OR “Chronically Ill*” [Title/Abstract] OR “Special Health Needs*” [Title/Abstract] OR “Special Health Care Needs*” [Title/Abstract] OR “Special HealthCare Needs*” [Title/Abstract] OR “Complex Chronic Ilness” [Title/Abstract] OR “Complex Chronic Condition*” [Title/Abstract] OR “Complex Chronic Disorder*” [Title/Abstract] OR “Chronic Condition*” [Title/Abstract]	287,625
#4	(“Health” [Mesh] OR “Delivery of health care” [Mesh] OR “Healthcare delivery” OR “Healthcare” OR “Health care”)	2,216,545
#5	#1 AND #2 AND #3 AND #4	373
Limited to language (English, Portuguese, Spanish)		364

MEDLINE (Pubmed) Survey conducted on 4 June 2021.

## Appendix B

**Table A2:** Data Extraction Instrument.

Data Extraction Tool	
**Review title:** Promotion of self-management of chronic illness in children and teenager: Scoping review.	
**Review purposes:** To analyze the existing literature and map the nature and extent of the intervention to promote self-management of chronic illness, implemented and evaluated in the context of health care provided to children and adolescents.	
**Question of investigation:** “What is the scientific evidence published about interventions that promote self-management of chronic disease in children and adolescents in the context of healthcare?”. **Sub-questions:** (1) What are the uses of the concept of self-management of chronic illness in children and adolescents in the context of healthcare? (2) What activities have been implemented and evaluated to promote self-management of children and adolescents in health care? (3) What are the characteristics of this intervention (unique and/or related activities, name, resources, frequency, duration and background)? (4) Which health professionals promote self-management in children and adolescents? (5) What outcomes were evaluated after the intervention?	
**Eligibility criteria:** - Participants: The review will consider studies that include school-age children and adolescents (between 6 and 19 years old). - Concept: Promotion of self-management, as a health intervention, in children and adolescents with chronic illness. - Context: Multidisciplinary studies will be included, in different areas of activity (hospital, primary care, among others). No cultural or geographic restrictions.	
**Details and characteristics of the study**	
Citation details (author/s, date, title, journal, volume, edition, pages)	
Country	
Background	
Participants (age/sex and sample size)	
Study design	
Details/results extracted from the evidence source	
Chronic disease	
Intervention	
Scientific Discipline	
Outcomes	
Limitations	
Future Perspectives	
Comments	

## Appendix C

**Table A3:** Characteristics of the articles included in this scope review.

Authors	Year	Country	Background	Type of Study	Population
Bagnasco et al. [32]	2015	Italy	Hospital (pre-discharge)	Mixed method–pilot study	17 teenagers with blood cancer
Buckner et al. [38]	2019	The United States of America	School	Quantitative study (quasi-experimental)	18 high school students with asthma (11 to 14 years old)
Cafazzo et al. [30]	2012	Canada	Diabetes clinic	Qualitative study (pilot study with ethnographic interviews)	20 adolescents with type 1 diabetes (12–16 years old), their families and diabetes care providers
Carroll et al. [47]	2014	India	Hospital endocrinology clinics	Qualitative Study (pilot study)	10 children (13 to 18 years old) with type I diabetes
Choi et al. [31]	2019	South Korea	Clinics for children with spina bifida	Quantitative study (quasi-experimental)	6 school-age children with spina bifida
Downs et al. [37]	2006	Australia	Home context	Randomized Controlled Study	43 children with cystic fibrosis and their main caregivers (6–11 years old)
Henkemans et al. [46]	2017	Netherlands	Department of Hospital Pediatrics	Quantitative Study (Randomized Controlled)	27 children with type 1 diabetes (7–14 years old)
Hommel et al. [34]	2020	The United States of America	Pediatric hospital	Qualitative Study (pilot study)	36 teenagers with migraine (11–18 years old)
Kew et al. [36]	2017	The United Kingdom	School, summer camp, primary care	Systematic review	1146 teenagers (11–17 years old)
Klaassen et al. [9]	2018	The United Kingdom and Netherlands	Department of Hospital and Home Pediatric Diabetes	Mixed method (Questionnaire and Interview)	21 adolescents (12–18 years old) with type 1 diabetes
Korus M et al. [27]	2015	Canada	Center for Transplantation and Hospital Regenerative Medicine	Qualitative Study (interview)	8 adolescents (12 and 17 years old) with kidney transplantation
Longacre et al. [44]	2018	The United States of America	Hospital Pediatric Cystic Fibrosis Center	Retrospective, descriptive and qualitative case study	60 pediatric patients with cystic fibrosis (6–18 years old)
Malheiro et al. [28]	2019	Portugal	Holiday Camp	Pilot, descriptive, exploratory study, representing the qualitative component of a quasi-experimental	51 young people with spina bifida (10–18) and 30 parents/caregivers
McClure et al. [35]	2018	The United States of America	Primary school	Qualitative Study (pilot project)	Students with asthma (90), teachers (12) and parents (1)
Meade et al. [29]	2003	The United States of America	Pediatric hospital	Qualitative study-Experimental program	Children and adolescents with kidney transplantation (13, between 11 and 17 years old)
Meyer et al. [33]	2021	Israel	Health communication groups with celiac disease	Quantitative Experimental Study	13 adolescents with celiac disease (13 and 18 years old)
Rhee et al. [39]	2012	The United States of America	Holiday camp	Quantitative Study (Randomized Controlled)	91 adolescents with asthma (13–17 years old)
Runge et al. [40]	2006	Germany	Specialized clinics and practices, as well as asthma clinics in hospitals	Quantitative study	438 asthmatic patients (8 to 16 years-old)
Schneider et al. [45]	2019	The United States of America	Pediatric Clinics	Exploratory Qualitative Study	20 teenagers with asthma (12 to 17 years old)
Stinson et al. [43]	2016	Canada	Rheumatology clinic in a large urban Canadian pediatric tertiary hospital	Quantitative Study (Randomized Controlled)	30 adolescents juvenile ideopathic arthritis (12–18 years old)
Tieffenberg et al. [2]	2000	Argentina	School and Community	Quantitative Study (Randomized Controlled)	355 school-age children between 6 and 15 years old
Van der Meer [41]	2007	Netherlands	Medical Center Department of Pediatrics Offices	Quantitative Study (Observational)	35 teenagers with asthma between 12 and 17 years old
Wiecha et al. [42]	2015	The United States of America	Community health centers	Qualitative Study (pilot)	58 children and adolescents with asthma (9 and 17 years old)

**Table A4:** Use of the concept of self-management of chronic illness in children and adolescents in the context of health care.

Author	Year	Title of Study	Page	Background	Concept	Consequent
Bagnasco et al. [32]	2015	Investigating the Use of Barrows Cards to Improve Self-Management and Reduce Health Costs in Adolescents with Blood Cancer: A Pilot Study	755	Lack of knowledge about therapeutic interventions	“Self-management aims to help patients maintain well-being (Lorig & Holman 2003) (…) it is based on problems, because it is based on the perception that patients have of the problems related to their conditions.” Corbin and Strauss (1988)	- Safety in therapeutic management
Korus et al. [27]	2015	Usability Test for Teenagers Gaining Control: “Manage My Transplant Online”	108 e 113	Difficulties in therapeutic management, post-transplant	“the individual’s ability to manage symptoms, treatment, physical and psychological consequences, and lifestyle changes inherent in living with a chronic illness” (Barlow et al., 2002)	- Prevent effects of incorrect therapeutic management, such as seizures - Avoiding readmissions to health services
Meade et al. [29]	2003	A self-management program for children and adolescents with kidney transplantation	165		“performance of therapeutic health care activities, often in collaboration with health professionals.” “ (Holroyd & Creer, 1986, p. xx). It involves the patient’s active participation in the management of their disease, becoming a member of the health care team. The basic skills of self-management include self-monitoring, medication compliance, environmental control, relaxation, and problem solving.	
Meyer et al. [33]	2021	Mobile Application to Promote Gluten Free Diet Self-Management in Adolescents with Celiac Disease-Proof of Concept Study	1 e 2	Eating difficulties	“Self-management is the interaction of health behaviors and processes related to the tasks throughout life in which people engage to take care of themselves and live well with a chronic condition. It consists of a set of, making plans, being flexible, and solving problems to support learning, remembering, planning, and deciding” (World Health Organization, 2001)	- Adherence to changes in your lifestyle - skills, such as making decisions and maintaining daily needs

**Table A5:** Activities implemented and evaluated to promote self-management of children and adolescents in health care.

Author	Year	Title of Study	Activities Implemented
Bagnasco [32]	2015	Investigating the use of Barrows cards to improve self-management and reduce healthcare costs in adolescents with blood cancer: a pilot study	Use of the barrows cards method (a learning theory-based, problem-based educational intervention strategy that uses at least 15 personalized cards to teach participants how to deal with a specific problem
Buckner et al. [38]	2019	School-based, interprofessional, asthma self-management education program for high school students: a test of feasibility	Development of an asthma self-management educational program in schools
Cafazzo et al. [30]	2012	mHealth app project for self-management of type 1 diabetes in adolescents: a pilot study	Using an mHealth App for Adolescent Type 1 Diabetes Self-Management
Carroll et al. [47]	2014	The HealthPia GlucoPack™ Diabetes Phone: A Usability Study	Use of a prototype Diabetes Health Pia GlucoPack ™ monitoring system, which integrates a small blood glucose monitoring device into a mobile phone battery
Choi et al. [31]	2019	A 2-step integrative education and mHealth program for self-management in Korean children with spina bifida: a feasibility study	Development of a 2-step self-management program, including on-site integrative education and a mHealth intervention, for children with biliary spine
Downs et al. [37]	2006	Benefits of an education program on self-management of airway clearance treatments with aerosols for children with cystic fibrosis	Development of an education program for self-management
Henkemans et al. [46]	2017	Evaluation project of a personal robot, playing an educational self-management game for children with type 1 diabetes	Using a personal robot to provide diabetes self-management education through a game
Kew et al. [36]	2017	Lay-led and peer support interventions for adolescents with asthma (review)	Lay-led and peer support interventions for adolescents with asthma
Klaassen et al. [9]	2018	Design and evaluation of a generalized training and gamification platform for young patients with diabetes	Development of a Pervasive Coaching and Gamification platform for Young Diabetes Patients
Korus et al. [27]	2015	Usability Test for Teenagers Gaining Control: “Manage My Transplant Online”	Development of an Internet-based self-management program for young people with kidney transplants
Longacre et al. [44]	2018	Clinical Adoption of Health Technology at the Pediatric Cystic Fibrosis Center at Skåne University Hospital in Lund, Sweden	Development of an application-based patient support system designed to promote collaborative care and improve self-management among pediatric patients living with chronic conditions.
Malheiro et al. [28]	2019	Self-Management Educational Program for Teenagers with Spina Bifida: What Do Young People and Their Caregivers Have to Say?	Adaptation of a program to the pediatric population and implemented in adolescents with spina bifida
McClure et al. [35]	2018	Improving asthma management in the elementary school environment: an education and self-management pilot project	Implementation of Green Means Go, an asthma education, self-assessment and self-report program
Meade et al. [29]	2003	A Self-Management Program for Adolescents and Children with Kidney Transplants	Development and implementation of a self-management program designed to reverse non-adherence in adolescents with kidney transplantation.
Meyer et al. [33]	2021	Mobile application to promote self-management of the gluten-free diet in adolescents with celiac disease–Proof of concept study	Use of a mobile app, Plan My CDay, to promote self-management skills among young people with CD during adolescence
Rhee et al. [39]	2012	Evaluating a Peer-led Asthma Self-Management Program and Program Benefits for Adolescent Peer Leaders	Development of an Asthma Self-Management Program for Adolescent Peer Leaders
Runge et al. [40]	2006	Results of a web-based patient education program for children and adolescents with asthma	An Internet-based continuing education program as a complement to a standardized patient self-management program
Schneider et al. [45]	2019	I have most of my asthma under control and I know how it works: user perceptions of the self-management mobile app tailored for teenagers	Using a self-managed mobile app
Stinson et al. [43]	2016	The iPeer2Peer Program: a pilot randomized clinical trial in adolescents with juvenile idiopathic arthritis	Developing an Online Peer Tutoring Program
Tieffenberg et al. [2]	2000	A randomized field trial of a child-centred training model with conic disease	Development of a child-centred self-management training model
Van der Meer [41]	2007	Internet-based self-management offers an opportunity to gain better control of asthma in teenagers	Internet short message service as tools for self-management of chronic diseases
Wiecha et al. [42]	2015	Evaluation of a web-based asthma self-management system: a randomized controlled pilot trial	Development of an interactive and engaging website for children, promoting self-management

**Table A6:** Characteristics of interventions (unique and/or related activities, name, resources, frequency, duration, and background).

Author	Year	Title of Study	Characteristics of Interventions
Bagnasco [32]	2015	Investigating the Use of Barrows Cards to Improve Self-Management and Reduce Health Costs in Adolescents with Blood Cancer: A Pilot Study	Use a deck of cards of at least 15 cards specially designed to learn independently how to deal with a specific complex problem. Students can choose from a list of possible actions to solve a problem and in the sequence the student deems appropriate.
Buckner et al. [38]	2019	School-based Interprofessional Asthma Self-Management Education Program for High School Students: A Feasibility Test	Asthma assessments, individual coaching and group education were done over 5 sessions. The instruments used were the Childhood Asthma Control Test, the Asthma Responsibility Questionnaire, the Self-Efficacy Scale.
Cafazzo et al. [30]	2012	mHealth app project for self-management of type 1 diabetes in adolescents: a pilot study	An app with data presentation and decision support alerts warnings that integrate into the daily workflow of blood glucose testing in teenagers. For more proactive management.
Carroll et al. [47]	2014	The HealthPia GlucoPack™ diabetes phone: a usability study	The HealthPia GlucoPack ™ Diabetes Monitoring System with a small blood glucose monitoring device built into a mobile phone battery. The device consists of a strip sensor, analog circuit, microcontroller unit, communication interface and telephone input/output. By supporting self-care, this combination of resources has the potential to reduce parent-child conflicts over diabetes control.
Choi et al. [31]	2019	A 2-step integrative education program and mHealth for self-management in Korean children with spina bifida: a feasibility study	The on-site integrative education program for children with spina bifida was developed as a 4-h, 30-min course in 6 sessions. The intervention involved various teaching methods (including a lecture and discussion), laboratory exercises (using a human anatomy model), as well as role play and group activities (using a board game that addressed aspects of family life, school, friendship and themselves). Based on the content of the on-site integrative education program, a mobile app named “Bright Stars™” was developed. A content validity test examining all instruments used was conducted by a panel of 8 experts.
Downs et al. [37]	2006	Benefits of an education program on self-management of airway clearance treatments using aerosols for children with cystic fibrosis	Airways, a pen and paper program, which was completed by the caregiver and the child together at home over a 10-week period (10 chapters, each with about 20 min to complete and provide information and exercises child-friendly behavior). The assessment was carried out immediately before and after the intervention period, 6 and 12 months after. Knowledge from the disciplines of medicine, physiotherapy, psychology and education were integrated and presented using cognitive-behavioral strategies, based on the Social Learning Theory.
Henkemans et al. [46]	2017	Evaluation project of a personal robot to play an educational self-management game in children with type 1 diabetes	Children with diabetes mellitus, type I participated in a randomized controlled trial in which they played a diabetes mellitus self-management education game, namely a diabetes test, with a personal or neutral robot on 3 occasions in the clinic. Personalized robotic behavior was based on the theory of self-determination, focusing on children’s needs in terms of competence, relationships and autonomy. The determinants were pleasure, motivation and knowledge about diabetes. The child-robot interaction was observed, including the level of involvement.
Hommel et al. [34]	2020	Digital Therapeutic Self-Management Intervention in Adolescents with Migraine: Feasibility and Preliminary Effectiveness from the “migraine manager”	16 intervention modules: Participants completed an assessment before receiving the intervention. An algorithm was used to individually adapt the assignments of the treatment modules based on the patients’ self-management needs. It took place through a series of focus group sessions/interviews with key stakeholders, including patients, parents/educators, and headache doctors. Diary-Participants completed a diary during the 8-week intervention period.
Kew et al. [36]		Lay-led and peer support interventions for adolescents with asthma (review)	They identified 5 studies, including 1146 adolescents with asthma. Studies varied by design, duration (2.5 to 9 months), environment (school, camp primary care) and how peer support or lay-led sessions were provided. Asthma severity varied, as did the number of smokers. 3 studies used a program called Triple A (Adolescent Asthma Action), whereby older teens are trained to teach sessions for younger students; one of these studies tested the addition of a quit-smoking program; other peer support group sessions, including messages played through an mp3 player to encourage adherence; and the third compared a day camp on asthma led by colleagues with another led by nurses and doctors.
Malheiro et al. [28]	2019	Self-Management Educational Program for Teenagers with Spina Bifida: What Do Young People and Their Caregivers Have to Say?	It consists of 7 physical sessions, directed to 2 groups, with different themes and different educational strategies, for 7 days.
McClure et al. [35]	2018	Improving asthma management in the elementary school environment: an education and self-management pilot project	Over four months, the master’s degree students in nursing provided educational sessions in small groups to teach how to identify the symptoms of asthma. An approach with specific asthma education curricula developed for students, teachers and parents was used.
Meade et al. [29]	2003	A Self-Management Program for Adolescents and Children with Kidney Disease Transplantation	The program was divided into 8 1-h sessions, including patients and their families. The aim was to provide information, support and an opportunity to practice kidney transplant-related self-management skills.
Meyer et al. [33]	2021	Mobile Application to Promote Gluten Free Diet Self-Management in Adolescents with Celiac Disease-Proof of Concept Study	“Plan My C-Day,”-The main feature of this first version of the app is the simulation of the preparation to participate in events related to food outside the home. Three events were chosen for this purpose: eating out with friends, a meal on a family vacation, and a meal during a school trip.
Runge et al. [40]	2006	Results of a web-based patient education program for children and adolescents with asthma	Series of 2-h educational sessions (conducted between July 2001 and December 2002) where knowledge and self-management skills in asthma were strengthened. Patients were educated in various fields related to the disease. During the role play exercises, critical situations in the daily life of asthma patients were simulated in small groups and discussed.
Schneider et al. [45]	2019	I have most of my asthma under control and I know how it works: user perceptions of the self-management mobile app tailored for teenagers	Adolescents participated in a 3-month study. During this time, participants received 2 daily reminders that invited them to enter asthma symptoms and peak flow values. The app determined the participants’ asthma status based on their individual peak flow goal and reported symptoms. Asthma status was color coded in green (indicates 80–100% of the subject’s peak flow value), yellow (50–80%), and red (less than 50%). For each zone, the participant received a recommended action that followed their personal treatment plan.
Stinson et al. [43]	2016	The iPeer2Peer Program: a pilot study randomized clinical trial in adolescents with juvenile idiopathic arthritis	The iPeer2Peer program focuses on pairing a teenager living with Juvenile Idiopathic Arthritis with a trained youth who successfully manages the same disease. Peer mentors strived to act as positive role models to help reinforce self-management while providing essential social support to adolescents with juvenile idiopathic arthritis. Acting as a mentor to peers can also positively impact peers on their own self-efficacy and self-management skills.
Tieffenberg et al. [2]	2000	A randomized field study: A model for children with chronic illnesses (asthma and epilepsy)	The model, based on playful techniques, consists of 5 meetings of 8 to 10 families, with groups of children and parents held simultaneously, coordinated by specially trained teachers and outside the hospital environment. Children are empowered to take a leadership role in managing their health; parents learn to be facilitators; and physicians provide guidance, acting as advisors. Group activities include games, drawings, stories, videos and role plays. Children and parents were interviewed before and after the program. Medical and school records were forwarded for emergency and routine appointments.
Van der Meer [41]	2006	Internet-based self-management offers an opportunity to gain better asthma control in teenagers.	1-month observational study of lung function and Internet-based symptom monitoring. 97 adolescents with mild to moderate persistent asthma monitored asthma control at a designated website. After 4 weeks, 35 adolescents participated in 8 focus groups. All participants received a handheld electronic spirometer) and were trained to perform three maneuvers each morning before receiving medication and record peak expiratory flow values by entering them daily into a designated web application or via SMS for a period of 1 month. Participants received instant feedback messages with peak expiratory flow values expressed as a percentage of the best expected or personal value, respectively. Weekly, participants completed the Asthma Control Questionnaire. After the electronic monitoring study, eight focus group sessions lasting from one to one and a half hours were conducted.
Wiecha et al. [42]	2015	Evaluation of a web-based asthma self-management system: a randomized controlled pilot trial	BostonBreathes is an interactive, web-based asthma education, monitoring, and communication system designed to improve asthma care with three main goals: Improve adherence to asthma control medications among children with asthma through education, self-monitoring and rewards; improve teamwork among health professionals who care for children with asthma by providing a platform for communication.

**Table A7:** Health professionals who promote self-management in children and teenagers.

Author	Year	Study Title	Professionals
Bagnasco et al. [32]	2015	Investigating the Use of Barrows Cards to Improve Self-Management and Reduce Health Costs in Adolescents with Blood Cancer: A Pilot Study	Nurses
Buckner et al. [38]	2019	Evaluation of an asthma self-management system based on web: a pilot randomized controlled trial	Nursing and Physiotherapy Students/Interns/Teachers
Cafazzo et al. [30]	2012	The HealthPia GlucoPack™ Diabetes Phone: A Usability Study	Doctors
Carroll et al. [47]	2014	A 2-step integrative education program and mHealth for self-management in Korean children with spina bifida: a feasibility study	Doctors
Choi et al. [31]	2019	Benefits of an education program on self-management of airway clearance treatments for children with cystic fibrosis using aerosols	Pediatric nurses
Downs et al. [37]	2006	Evaluation project of a personal robot with a type 1 diabetes self-management educational game for children	Psychologist/Physiotherapist, Physician
Henkemans et al. [46]	2017	Digital Therapeutic Self-Management Intervention in Adolescents with Migraine: Preliminary Feasibility and Effectiveness of the “Migraine Manager”	Doctor/Nurse/Nutritionist Psychologist
Hommel et al. [34]	2020	Evaluation of a Peer-led Asthma Self-Management Program and Program Benefits for Adolescent Peer Leaders	Doctors
Kew et al. [36]	2017	Design and evaluation of a generalized training and gamification platform for young patients with diabetes	Nurses/Doctors/Peers
Malheiro et al. [28]	2019	Self-Management Educational Program for Teenagers with Spina Bifida: What Do Young People and Their Caregivers Have to Say?	Nurse/lay leaders
McClure et al. [35]	2018	Improving asthma management in the elementary school environment: an education and self-management pilot project	Nursing Students/Teachers
Meade et al. [29]	2003	A Self-Management Program for Adolescents and Children with Kidney Transplants	Nurse/Physician
Meyer et al. [33]	2021	Mobile Application to Promote Gluten Free Diet Self-Management in Adolescents with Celiac Disease–Proof of Concept Study	Occupational Therapy/Engineering and Industrial Management
Rhee et al. [39]	2012	Lay-led and peer support interventions for adolescents with asthma (review)	Nurses
Runge et al. [40]	2006	Results of a web-based patient education program for children and adolescents with asthma	Doctor
Schneider et al. [45]	2019	I have most of my asthma under control and I know how it works: user perceptions of the self-management mobile app tailored for teenagers	Doctor/Nutritionist
Stinson et al. [43]	2016	The iPeer2Peer Program: a pilot randomized clinical trial in adolescents with juvenile idiopathic arthritis	Doctors
Tieffenberg et al. [2]	2000	Internet-based self-management offers an opportunity to gain better control of asthma in adolescents.	Doctors
Van der Meer [41]	2006	School-based Interprofessional Asthma Self-Management Education Program for High School Students: A Feasibility Test	IT/Nurses/Doctors
Wiecha et al. [42]	2015	mHealth app project for self-management of type 1 diabetes in adolescents: a pilot study	Nurses/Doctors

**Table A8:** The results that were evaluated after the intervention.

Author	Year	Study Title	Topics Evaluated after the Intervention
Bagnasco et al. [32]	2015	Investigating the Use of Barrows Cards to Improve Self-Management and Reduce Health Costs in Adolescents with Blood Cancer: A Pilot Study	O método Barrows Cards melhorou significativamente a adesão à terapia imunossupressora e reduziu as readmissões. Poderá também reduzir significativamente os custos dos cuidados de saúde.
Buckner et al. [38]	2019	School-based Interprofessional Asthma Self-Management Education Program for High School Students: A Feasibility Test	The program’s community partnership approach, which included nursing and respiratory physiotherapy students, interns, and faculty, proved beneficial.
Cafazzo et al. [30]	2012	mHealth app project for self-management of type 1 diabetes in adolescents: a pilot study	The pilot evaluation showed that the mean daily frequency of blood glucose measurement increased by 50%. Satisfaction was high, with 88% of respondents saying they would continue using the system.
Carroll et al. [47]	2014	The HealthPia GlucoPack™ Diabetes Phone: A Usability Study	The teenagers liked the integration of the two technologies and agreed that the glucometer was easy to use and that the tool was useful in managing diabetes.
Choi et al. [31]	2019	A 2-step integrative education program and mHealth for self-management in Korean children with spina bifida: a feasibility study	All children realized that this program was usable and viable to maintain self-management behavior. A statistically significant difference was observed in the domain of children’s self-care behavior between the first and second post-test.
Downs et al. [37]	2006	Benefits of an education program on self-management of airway clearance treatments for children with cystic fibrosis using aerosols	The intervention group increased the percentage of prescribed aerosols taken and this fact remained in the following 12 months. Children in the intervention group they increased their knowledge and this was maintained for the next 12 months. They reported feeling more positive about their treatment immediately after the intervention. There were no significant changes between the control group for these variables over time. The positive results suggest that ‘Airways’ is a valuable educational tool for primary school-age children and their caregiver.
Klaassen et al. [29]	2018	A self-management program for children and adolescents with kidney transplantation	The program was effective in creating a supportive environment for both patients and their parents in responding to health concerns.
Malheiro et al. [28]	2019	Self-Management Educational Program for Teenagers with Spina Bifida: What Do Young People and Their Caregivers Have to Say?	Improvements were observed in the self-management skills of the youngsters, who demonstrated responsibility, proactivity, confidence, problem-solving capacity and autonomy. Of the psychoeducational strategies, the emphasis was on the technique of problem solving, dramatization, videos, peer tutoring and modeling. Changes in young mentors also reveal the effectiveness of this strategy.
McClure et al. [35]	2018	Improving asthma management in the elementary school environment: an education and self-management pilot project	Previously, no students performed daily self-assessments. By the end of the program, all students have accurately identified asthma symptoms and action plans. Teachers reported greater knowledge about asthma. In the current climate of school nurse shortages, school self-management of asthma episodes can be improved with partnerships between elementary and high schools.
Meyer et al. [33]	2021	Mobile Application to Promote Gluten Free Diet Self-Management in Adolescents with Celiac Disease-Proof of Concept Study	The contents, features and functions of the Plan My C-Day app operated well and the simulations were easy to understand and complete.
Runge et al. [40]	2006	Results of a web-based patient education program for children and adolescents with asthma	The program offers the potential to lessen symptoms. Subgroup analysis showed that, within 1 year, savings exceed intervention costs in patients with moderate or severe asthma.
Schneider et al. [45]	2019	I have most of my asthma under control and I know how it works: user perceptions of the self-management mobile app tailored for teenagers	Participants found the application functional and easy to use. Most expressed that the application helped them to self-manage their asthma through asthma status tracking and text reminders to regularly test peak flow. They suggested some improvements to make it more captivating and appealing.
Stinson et al. [43]	2016	The iPeer 2 Peer Program: a pilot randomized clinical trial in adolescents with juvenile idiopathic arthritis	The main results focused on implementation (feasibility and acceptability). Secondary outcomes focused on effectiveness (measures of self-management, self-efficacy, pain, social support and quality of life). Participants demonstrated improvements in their self-management skills compared to the control group.
Van der Meer et al. [41]	2006	Internet-based self-management offers an opportunity to gain better control of asthma in adolescents.	The first group (Patients with poor asthma control) revealed the following benefits of self-management of asthma via the Internet: viable electronic monitoring; easily accessible information; email communication; and use of electronic action plan. Personal benefits included the ability to react to changes and optimize asthma control. In patients with good control, no benefits were identified.
Wiecha et al. [42]	2015	Evaluation of an asthma self-management system based onweb: a pilot randomized controlled trial	After 6 months, reported wheezing improved significantly in the intervention and control groups, and there were significant improvements in the intervention group only in nocturnal awakening and parental sleep loss in the intervention group. Among the subgroup of individuals with poor adherence to control medication at baseline, adherence improved significantly only in the intervention group.
